# Clinical features, management, and long-term visual outcomes of glaucoma associated with cosmetic iris implants

**DOI:** 10.1038/s41433-026-04416-1

**Published:** 2026-03-16

**Authors:** Kasem Seresirikachorn, Ta Chen Peter Chang

**Affiliations:** 1 Department of Ophthalmology, College of Medicine, Rangsit University, Rajavithi Hospital, Bangkok, Thailand; 2 Bascom Palmer Eye Institute, University of Miami Miller School of Medicine, Miami, FL USA

**Keywords:** Glaucoma, Education

## Introduction

Cosmetic iris implants, used for non-medical eye colour change, have been associated with severe complications, especially glaucoma, affecting about 40% of eyes and often requiring surgery [1]. Long-term outcomes of glaucoma management remain unclear. This study investigates glaucoma characteristics and treatment results, offering insights for ophthalmologists and raising awareness among potential patients.

## Methods

This retrospective study, approved by the Bascom Palmer Eye Institute Institutional Review Board, surveyed consecutive glaucoma cases following cosmetic iris implantation (2014–2023). Demographic, clinical, and treatment data were analysed. Success was defined as intraocular pressure 5–21 mmHg with ≥20% reduction from baseline without re-operation; “complete success” required no medication, while “qualified success” required medication. Failure was the absence of success on two consecutive visits or re-operation. Outcomes were assessed using Kaplan-Meier survival analysis and standard statistical tests.

## Results

Nine patients with bilateral glaucoma following cosmetic iris implantation were included (Table 1). The mean age at implant surgery was 28.3 ± 5.8 years, and glaucoma was diagnosed on average 3.7 ± 3.3 years after the implant procedure. Most implants were performed in India and Panama.

**Table 1:** Clinical characteristics and procedures in glaucoma associated with cosmetic iris implants.

Patient Characteristics	Number of Patients (*N* = 9), *N* (%)
Male, *N* (%)	5 (55.5%)
Race	
- White, Hispanic, *N* (%)	7 (77.8%)
- White, Non-Hispanic, *N* (%)	2 (22.2%)
Age at iris implant, years mean ± SD; median (range)	28.3 ± 5.8; 28 (21–40)
Age at glaucoma-associated iris implant, years mean ± SD; median (range)	31.8 ± 5.6; 31 (24–42)
Time between iris implant and glaucoma diagnosis, years mean ± SD; median (range)	3.7 ± 3.3; 2 (0–9)
Follow-up time between glaucoma diagnosis and last visit, years mean ± SD; median (range)	5.6 ± 3.5; 5 (2–12)
**Location of iris implant surgery**	
- India, *N* (%)	2 (22.22%)
- Panama, *N* (%)	2 (22.22%)
- Mexico, *N* (%)	1 (11.11%)
- Colombia, *N* (%)	1 (11.11%)
- Venezuela, *N* (%)	1 (11.11%)
- Unknown, *N* (%)	2 (22.22%)
**Type of iris implant surgery**	
-Bright Ocular Iris	3 (33.33%)
-Unknown	6 (66.67%)
**List of Procedures**	**Number of Eyes (N** = **18), N (%)**
**Iris implant removal**	16 (88.9%)
**Cataract surgery**	11 (61.1%)
**Primary Glaucoma Treatment**	
Glaucoma Drainage Device (Baerveldt Glaucoma Implantation-350)	7 (38.9%)
Glaucoma Drainage Device (Ahmed-FP7)	5 (27.8%)
Glaucoma medication only	4 (22.2%)
Trabeculectomy	2 (11.1%)
**Required second glaucoma surgery**	
Glaucoma Drainage Device (Baerveldt Glaucoma Implantation-350)	4 (22.2%)
Cyclophotocoagulation	2 (11.1%)
**First Corneal Transplantation**	
Descemet’s Stripping Endothelial Keratoplasty (DSEK)	6 (33.3%)
Penetrating Keratoplasty procedure (PKP)	2 (11.1%)
Descemet Stripping Automated Endothelial Keratoplasty (DSAEK)	1 (5.6%)
**Repeat corneal transplantation**	
Penetrating Keratoplasty procedure (PKP)	4 (22.2%)

Baerveldt-350 (Johnson & Johnson, New Brunswick, NJ, the United States of America).

Ahmed-FP7 (New World Medical, Inc., Rancho Cucamonga, CA, the United States of America).

Out of 18 eyes, 16 (88.9%) underwent iris implant removal. Among these, 61.1% required cataract surgery. The most common primary treatment for glaucoma was the implantation of a glaucoma drainage device (GDD), used in 66.7% of cases, with a mean surgical age of 33.4 ± 5.9 years. Specifically, the Baerveldt-350 (38.9%), followed by the Ahmed-FP7 (27.8%). Other treatments included glaucoma medications alone (22.2%) and trabeculectomy (11.1%).

Additionally, 33.3% of eyes required a second glaucoma procedure at a median of 9 months following the initial surgery, either another GDD or cyclophotocoagulation. Corneal transplantation was necessary in 50% of eyes, with Descemet-stripping endothelial keratoplasty being the most common type, performed in 33.3% of cases.

**Fig. 1:**
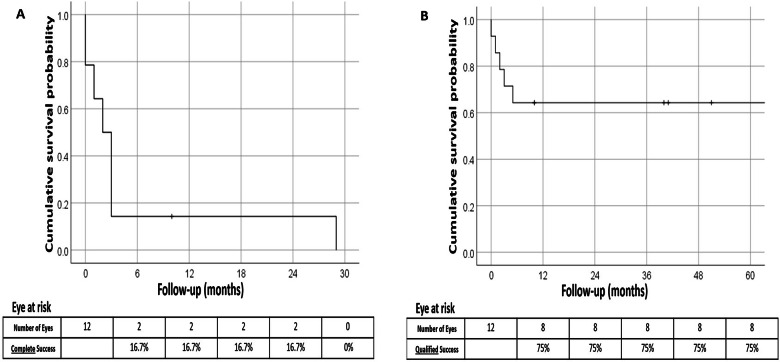
Kaplan–Meier survival curves following glaucoma drainage device implantation in patients with glaucoma associated with cosmetic iris implants. **A** Cumulative probability of complete success, (**B**) Cumulative probability of qualified success.

The cumulative probability of complete surgical success following GDD implantation in patients with glaucoma related to cosmetic iris implants was 16.7% at 1 year. However, by the third postoperative year, no cases achieved complete success (Fig. 1A). In contrast, the rate of qualified surgical success remained stable at 75% at 1, 3, and 5 years postoperatively (Fig. 1B). Two eyes that underwent trabeculectomy eventually required GDD due to uncontrolled IOP.

Despite intraocular pressure reduction, cup-to-disc ratio increased significantly (*p* = 0.003), and blindness (based on the World Health Organization criteria) rose from 3 eyes at presentation to 6 at final follow-up.

## Discussion

In our cohort, despite undergoing glaucoma surgery, many patients continued to rely on anti-glaucoma medications to maintain IOP control, and several had disease progression despite aggressive interventions. The most commonly performed surgery was implantation of a GDD, consistent with previous studies [2]. While complete success was rare, most eyes achieved qualified success throughout the five-year follow-up.

In conclusion, glaucoma secondary to cosmetic iris implants often requires surgical management, with GDD achieving a qualified success rate of 75% at five years. These findings underscore the importance of public awareness and education regarding elective cosmetic iris implant procedures, particularly in countries where such implants are more commonly performed.

## Data Availability

The datasets generated during and/or analysed during the current study are available from the corresponding author on reasonable request.
